# Attrition in the Gothenburg H70 birth cohort studies, an 18-year follow-up of the 1930 cohort

**DOI:** 10.3389/fepid.2023.1151519

**Published:** 2023-05-09

**Authors:** Lina Rydén, Hanna Wetterberg, Felicia Ahlner, Hanna Falk Erhag, Pia Gudmundsson, Xinxin Guo, Erik Joas, Lena Johansson, Silke Kern, Madeleine Mellqvist Fässberg, Jenna Najar, Mats Ribbe, Therese Rydberg Sterner, Simona Sacuiu, Jessica Samuelsson, Robert Sigström, Johan Skoog, Margda Waern, Anna Zettergren, Ingmar Skoog

**Affiliations:** ^1^ Neuropsychiatric Epidemiology Unit, Department of Psychiatry and Neurochemistry, Institute of Neuroscience and Physiology, Sahlgrenska Academy at the University of Gothenburg, Mölndal, Sweden; ^2^ Centre for Ageing and Health (AgeCap) at the University of Gothenburg, Gothenburg, Sweden; ^3^ Department of Mood Disorders, Region Västra Götaland, Sahlgrenska University Hospital, Gothenburg, Sweden; ^4^ Psychiatry, Cognition and Old Age Psychiatry Clinic, Region Västra Götaland, Sahlgrenska University Hospital, Gothenburg, Sweden; ^5^ Aging Research Center, Department of Neurobiology, Care Sciences and Society, Karolinska Institutet and Stockholm University, Stockholm, Sweden; ^6^ Region Västra Götaland, Department of Psychiatry, Psychotic Disorders, Sahlgrenska University Hospital, Mölndal, Sweden

**Keywords:** attrition, population studies, drop out, epidemiogy, representativeness

## Abstract

**Background:**

Longitudinal studies are essential to understand the ageing process, and risk factors and consequences for disorders, but attrition may cause selection bias and impact generalizability. We describe the 1930 cohort of the Gothenburg H70 Birth Cohort Studies, followed from age 70 to 88, and compare baseline characteristics for those who continue participation with those who die, refuse, and drop out for any reason during follow-up.

**Methods:**

A population-based sample born 1930 was examined with comprehensive assessments at age 70 (*N* = 524). The sample was followed up and extended to increase sample size at age 75 (*N* = 767). Subsequent follow-ups were conducted at ages 79, 85, and 88. Logistic regression was used to analyze baseline characteristics in relation to participation status at follow-up.

**Results:**

Refusal to participate in subsequent examinations was related to lower educational level, higher blood pressure, and lower scores on cognitive tests. Both attrition due to death and total attrition were associated with male sex, lower educational level, smoking, ADL dependency, several diseases, poorer lung function, slower gait speed, lower scores on cognitive tests, depressive symptoms, and a larger number of medications. Attrition due to death was also associated with not having a partner.

**Conclusions:**

It is important to consider different types of attrition when interpreting results from longitudinal studies, as representativeness and results may be differently affected by different types of attrition. Besides reducing barriers to participation, methods such as imputation and weighted analyses can be used to handle selection bias.

## 1. Introduction

Longitudinal population studies are essential to study ageing processes and incidence, risk factors, and consequences of disorders. However, differences in characteristics between participants and those who drop out can affect representativeness compared to the target population (1) and impact results in association studies (2). In attrition analyses, it is essential to differentiate between various types of attrition, especially to separate attrition due to death and non-death attrition since their impact on representativeness and study results differ (1). Non-death attrition (e.g. refusal to participate and contact failure) may be more important for representativeness than attrition due to death since deaths occur both in the target population and the study cohort (1). All types of attrition may introduce bias and impact effect estimates in association studies, but attrition due to death is especially relevant in longitudinal studies involving older adults where death rates are high (2).

The Gothenburg H70 Birth Cohort Studies (the H70 studies) are multidisciplinary, population-based studies of older adults in Gothenburg, Sweden, aiming to study prevalence, incidence, risk factors, and consequences of physical and mental disorders (3). The H70 studies started in 1971, with baseline examinations of 70-year-olds born 1901-02. Since then, five birth cohorts with baseline at age 70 have been examined longitudinally. Since the start, more than 700 papers have been published using H70 data and the longitudinal design has e.g. enabled the discovery of several risk factors for dementia (4–9) and depression (7, 10). The consecutive recruitment of new birth cohorts of the same age and the use of similar examinations has enabled studies of time trends, and its effect on risk factors and outcomes (11–13).

The aim of this study is to examine if individuals characteristics at age 70 and 75 in the 1930 cohort of the H70 studies, differ between those who participate and those who refuse, die, or drop out for any reason at each follow-up.

## 2. Methods

All samples from the H70 Studies are systematically selected from the Swedish Population Register based on birth dates to yield representative samples. The five first cross-sectional samples of the 1930-cohort from year 2000–02 to 2018–19 have been described in detail previously (14).

Part of the sample born 1930 (i.e. women born on day 6, 12, 18, 24 and 30 of each month who lived in Gothenburg at the time of the invitation) has been examined previously within the Prospective Population Study of Women (the PPSW study) that started in 1968–69 and was followed-up in 1974–75, 1980–81, and 1992–94 (15). When inviting individuals in year 2000–02 to the first examination of the 1930 cohort within the H70 study, the sample from the PPSW study was extended and included both male and female residents in Gothenburg born on day 3, 6, 12, 18, 21, 24 and 30 of each month (except for women born on day 21, were only those born in January-July were included). At the first follow-up of the H70 1930 cohort in year 2005, the sample was further extended to include male and female residents in Gothenburg born on days 2, 3, 5, 6, 11, 12, 16, 18, 20, 21, 24, 27, or 30 of each month (except for day 27, were only those born in January-May were included).

A letter was first sent to all sampled individuals, who thereafter were contacted by telephone and asked about participation. If they could not visit the outpatient clinic, they were offered home visits. Exclusion criteria included emigration before examination start, inability to speak the Swedish language (language difficulties) and contact failure. The baseline participants were contacted again at each follow-up, except for those who wished not to be contacted further.

### 2.1. Description of the sample with baseline at age 70

In 2000–02, 775 70-year-olds were invited. Among those, 12 could not participate due to language difficulties, five died before the examination, four could not be contacted, and one emigrated before the examination, leaving an eligible sample of 753 (390 women, 363 men). Of these, 524 (281 women, 243 men) accepted to participate (response rate 70%), while 229 declined participation. A total of 173 participants had previously been examined before age 70 as part of the PPSW study.

### 2.2. Description of the sample with baseline at age 75

In 2005–07, 1250 75-year-olds were invited. Among those, 24 could not participate due to language difficulties, 11 died before the examination, 17 could not be contacted, and two had emigrated, leaving an eligible sample of 1196 (684 women, 512 men). Of these, 767 (438 women, 329 men) accepted to participate (response rate 64%), while 429 declined participation. A total of 386 participants had previously been examined at age 70 and 116 had been examined before age 70 as part of the PPSW study.

### 2.3. Data collection procedures

The baseline examinations at age 70 and 75 included semi-structured somatic, psychiatric, dietary (at age 70 only), functional, and social interviews, as well as questions about medications. In addition, physical examinations (e.g., anthropometry, blood pressure, ECG, spirometry, gait speed, and grip strength), and tests of cognition and personality were performed. Biomarkers included blood sampling, genetic analyses, bioimpedance, and computed tomography of the brain (at age 70 only). All examinations are described in detail elsewhere (3). The characteristics examined in the attrition analyses are defined as follows:

***Having a partner*** was defined as being married or cohabitant or having a partner but living separately. ***Educational level*** was dichotomised as having mandatory education (corresponding to 7 years) or less vs. more than mandatory education. ***Smoking*** was dichotomized as being a current smoker vs. past or never smoker. ***Alcohol risk consumption*** was defined according to the NIAAA guidelines as >98 g alcohol/week (16) and was based on self-reported alcohol consumption during the last month. Height and weight were measured, and ***body mass index (BMI)*** was calculated.

***Blood pressure*** was measured with a manual sphygmomanometer in the right arm after five minutes' rest in a seated position. ***Peak Expiratory Flow (PEF)*** was measured with a Peak Flow Meter in liter per minute and calculated as percent of expected value based on sex, height, and age according the equation suggested by Hankinson et al. (17, 18). Self-selected indoor ***gait speed*** (30 meter in 2000 and 20 meter in 2005) with a standing start was measured in meters per second. ***Activities of daily living (ADL)*** was assessed according to the Katz Index of Independence in Activities of Daily Living (ADL) (19, 20), using six domains (bathing, dressing, toileting, transferring, continence, and feeding), and the Lawton Instrumental Activities of Daily Living (IADL) scale (21), where four domains were assessed (housekeeping, shopping, mode of transportation, and food preparation). The participants were classified as ADL/IADL dependent if dependent in at least one ADL or IADL domain. The total ***number of medications*** was recorded. ***Myocardial infarction*** was identified from self-reports, the National Patient Register (NPR) [International Classification of Diseases (ICD) 8-SE codes 410, 412.01, 412.09; ICD9-SE codes 410, 411A, 411C, 412; ICD10-SE codes I21-I23, I24.1, I25.2, I25.6, U98], or presence of major or intermediate Q-waves on ECG [Minnesota code (MC) 1-1-X or 1-2-X, excluding 1-2-6 and 1-2-8] (22). ***Atrial fibrillation or flutter*** was identified from close-informant interviews (in 2000), self-reports (in 2005), the NPR (ICD-8-SE code 427.92; ICD-9-SE code 427D; ICD-10-SE code I48), or ECGs (MC 8-3). ***Heart failure*** was identified from the NPR (ICD-8-SE code 427.00; ICD-9-SE code 428; ICD-10-SE code I11.0, I13.0, I13.2, I50). ***Diabetes mellitus*** was defined as present treatment with insulin or antidiabetic medications. ***Treatment for hypertension and hypercholesterolemia*** were defined as self-reported use of antihypertensive or lipid-lowering medication. ***Stroke*** was identified from self-reports and close-informant interviews, the NPR (ICD8-SE codes 431, 433, 434; ICD9-SE codes 431, 432, 434, 438; ICD10-SE codes I61-I63, I69.1-I69.4), and hospital medical records. ***Dementia*** was based on the Diagnostic and Statistical Manual of Mental Disorders, third edition revised (DSM-III-R), using combined information from neuropsychiatric examinations and close-informant interviews, as described in detail previously (6, 23, 24). ***The Montgomery-Åsberg Depression Rating Scale (MADRS)*** (25) was used to assess depressive symptoms and depression severity. Blood was drawn and DNA was extracted according to standard procedures. ***APOE*** genotyping was performed by KASPar® PCR SNP genotyping system (LGC Genomics, Hoddesdon, Herts, UK) or by mini-sequencing, as previously described in detail (26). Genotype data for the SNPs rs7412 and rs429358 were used to define *ɛ2*, *ɛ3*, and *ɛ4* alleles. The cognitive tests included ***word fluency*** (name as many animals as possible in one minute), and a ***free recall*** test (repeat 12 shown objects after distraction).

### 2.4. Attrition during follow-up

The baseline participants were classified as participant or drop-out at each follow-up. Those who dropped out were classified as refusals, deceased, or other reasons for attrition, including contact failure, emigration, language difficulties, and technical reasons. Total attrition includes all reasons for attrition. Death dates were obtained from the Swedish Tax Agency.

### 2.5. Statistical analyses

Logistic regression was used to analyze associations between characteristics at ages 70 and 75 and attrition at each follow-up. Separate analyses were performed for each characteristic (as the predictor variable) in relation to three different types of attrition (as the outcome variable, i.e., refusal, death, and total attrition) compared to participation at each follow-up. Attrition due to death included the cumulative deaths from baseline until the specific examination.

First, unadjusted analyses were performed. Second, adjusted analyses were performed, including sex and education as potential covariates. Third, sensitivity analyses were performed for analyses including cognitive level where individuals with baseline dementia were excluded. No corrections for multiple testing were performed since avoiding type II errors (i.e., to fail to reject a null-hypothesis that is false) were regarded more important than avoiding type I errors (i.e., to reject a null-hypothesis that is actually true). A *p*-value < 0.05 (two-tailed) was considered statistically significant. Analyses were conducted in SPSS, version 29.0.

### 2.6. Ethics

This study was performed in line with the principles of the Declaration of Helsinki. Approval was granted by the Regional Ethics Committee for Medical Research at the University of Gothenburg. Informed consent was obtained from the participants when possible. In cases where informed consent was not possible to obtain from the participant (e.g., due to dementia), informed consent was obtained from a close relative.

## 3. Results

Baselines characteristics at age 70 and 75, stratified by sex, are shown in Table 1.

**Table 1:** Sample characteristics at age 70 and 75, by sex.

	Age 70 Total group *N* = 524		Men *N* = 243		Women *N* = 281		Age 75 Total group *N* = 767		Men *N* = 329		Women *N* = 438	
	n/N	(%)	n/N	(%)	n/N	(%)	n/N	(%)	n/N	(%)	n/N	(%)
Women	281/524	(53.6)	–	–	–	–	438/767	(57.1)	–	–	–	–
Having partner	345/510	(67.6)	202/242	(83.5)	143/281	(53.4)	464/753	(61.6)	258/326	(79.1)	206/427	(48.2)
More than mandatory education	234/517	(45.3)	110/241	(45.6)	124/276	(44.9)	376/763	(49.3)	170/328	(51.8)	206/435	(47.4)
Current smoker	80/510	(15.7)	36/239	(15.1)	44/271	(16.2)	95/752	(12.6)	43/325	(13.2)	52/427	(12.2)
Alcohol risk consumption	72/461	(15.6)	54/216	(25.0)	18/245	(7.3)	119/637	(18.7)	80/277	(28.9)	39/360	(8.9)
ADL dependent	55/477	(11.5)	24/226	(10.6)	31/251	(12.4)	97/691	(14.0)	29/300	(9.7)	68/391	(17.4)
Myocardial infarction	57/524	(10.9)	39/243	(16.0)	18/281	(6.4)	102/767	(13.3)	65/329	(19.8)	37/438	(8.4)
Atrial fibrillation	56/524	(10.7)	41/243	(16.9)	15/281	(5.3)	90/767	(11.7)	57/329	(17.3)	33/438	(7.5)
Heart failure	18/524	(3.4)	11/243	(4.5)	7/281	(2.5)	35/767	(4.6)	16/329	(4.9)	19/438	(4.3)
Treatment for diabetes	42/522	(8.0)	23/243	(9.5)	19/279	(6.8)	92/767	(12.0)	46/329	(14.0)	46/438	(10.5)
Treatment for hypertension	138/514	(26.8)	57/240	(23.8)	81/274	(29.6)	276/736	(37.5)	115/317	(36.3)	161/419	(38.4)
Stroke	27/524	(5.2)	11/243	(4.5)	16/281	(5.7)	77/767	(10.0)	41/329	(12.5)	36/438	(8.2)
Dementia	15/499	(3.0)	5/229	(2.2)	10/270	(3.7)	45/758	(5.9)	22/325	(6.8)	23/433	(5.3)
APOE e4	146/506	(28.9)	66/239	(27.6)	31/251	(12.4)	207/730	(28.4)	90/318	(28.3)	117/412	(28.4)
	**mean ** **±** ** SD**	**(** **N)**	**mean ** **±** ** SD**	**(** **N)**	**mean ** **±** ** SD**	**(** **N)**	**mean ** **±** ** SD**	**(** **N)**	**mean ** **±** ** SD**	**(** **N)**	**mean ** **±** ** SD**	**(** **N)**
BMI (kg/m^2^)	27.0 ± 4.2	(510)	27.0 ± 3.9	(242)	27.0 ± 4.5	(268)	26.7 ± 4.3	(740)	26.8 ± 3.6	(320)	26.6 ± 4.7	(420)
SBP (mmHg)	155 ± 22	(520)	156 ± 20	(243)	153 ± 23	(277)	151 ± 21	(763)	151 ± 21	(329)	150 ± 22	(434)
DBP (mmHg)	84 ± 11	(520)	85 ± 10	(243)	83 ± 11	(277)	81 ± 10	(763)	82 ± 11	(329)	80 ± 10	(434)
PEF (% of expected)	96 ± 24	(488)	95 ± 25	(235)	97 ± 23	(253)	108 ± 26	(693)	110 ± 29	(307)	107 ± 24	(386)
Gait speed (m/s)	1.29 ± 0.22	(411)	1.33 ± 0.2	(206)	1.25 ± 0.22	(205)	1.18 ± 0.19	(574)	1.21 ± 0.18	(248)	1.15 ± 0.20	(326)
Word fluency	22.6 ± 6.9	(488)	23 ± 6.6	(228)	22.3 ± 7.2	(260)	20.6 ± 6.8	(718)	20.5 ± 6.7	(308)	20.7 ± 6.8	(410)
Free recall	7.0 ± 1.9	(486)	6.6 ± 1.8	(226)	7.4 ± 2.0	(260)	7.1 ± 2.1	(719)	6.6 ± 2.1	(307)	7.4 ± 2.1	(412)
	**median [IQR],**	**(** **N)**	**median [IQR],**	**(** **N)**	**median [IQR],**	**(** **N)**	**median [IQR],**	**(** **N)**	**median [IQR],**	**(** **N)**	**median [IQR],**	**(** **N)**
Number of medications	2.5 [1–5],	(522)	2 [1–4],	(243)	3 [1–5],	(279)	4 [2–6],	(747)	3 [1–6],	(320)	4 [2–6],	(427)
MADRS	3 [1–6],	(480)	3 [0–5],	(228)	3 [1–7],	(252)	4 [2–9]	(711)	4 [1–8]	(300)	5 [2–10]	(411)

ADL, Activities of Daily Living; APOE, Apolipoprotein E; BMI, Body Mass Index; SBP, Systolic Blood Pressure; DBP, Diastolic Blood Pressure; PEF, Peak Expiratory Flow; MADRS, Montgomery Åsberg Depression Rating Scale.

### 3.1. Follow-up from age 70

**Figure 1:**
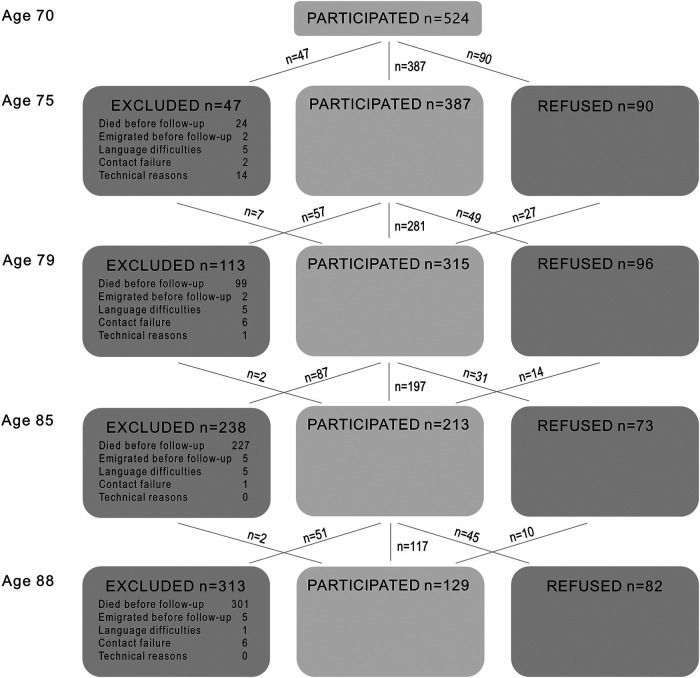
Longitudinal sample with baseline at age 70 in 2000–02.

Table 2 shows the number and proportion of individuals who participated and refused participation at each follow-up, died before each follow-up, or dropped out for other reasons (i.e., contact failure, emigration, language difficulties, or technical reasons). In total, 97 individuals participated once, 110 participated twice, 125 participated three times, 84 participated four times, and 108 participated five times. Among the 524 who participated at baseline, 427 (81%) participated in at least one follow-up examination. The response rate among survivors was above 70% at all follow-ups, except at age 88, where the response rate among survivors was 58%. A flow chart of the sample is found in Figure 1.

**Table 2:** The proportion of individuals who participated and dropped out compared to baseline.

Baseline at age 70						Baseline at age 75				
Age	Participated N (%)	Refused N (%)	Died N (%)	Other^a^ N (%)	RR among survivors	Participated N (%)	Refused N (%)	Died N (%)	Other^a^ N (%)	RR among survivors
70	524 (100)	–	–	–	–	–	–	–	–	–
75	387 (74)	90 (17)	24 (5)	23 (4)	77%	767 (100)	–	–	–	–
79	315 (60)	96 (18)	99 (19)	14 (3)	74%	520 (68)	129 (17)	98 (13)	20 (3)	78%
85	213 (41)	73 (14)	227 (43)	11 (2)	72%	354 (46)	109 (14)	291 (38)	13 (2)	74%
88	129 (25)	82 (16)	301 (57)	12 (2)	58%	215 (28)	137 (18)	402 (52)	13 (2)	59%

RR, Response Rate.

^a^ Emigration, language difficulties, contact failure, and technical reasons.

### 3.2. Follow-up from age 75

**Figure 2:**
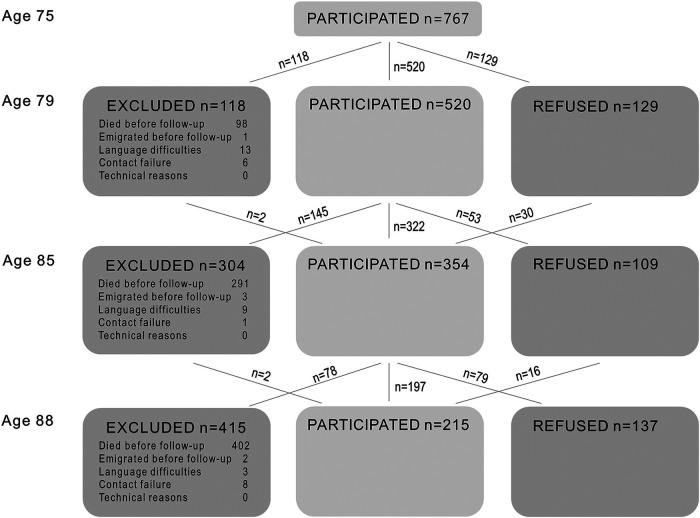
Longitudinal sample with baseline at age 75 in 2005-07.

Table 2 shows the number and proportion of individuals who participated and refused at each follow-up, died before each follow-up, or dropped out for other reasons. In total, 208 individuals participated once, 216 participated twice, 156 participated three times, and 187 participated four times. Among the 767 who participated at age 75, 559 (73%) participated in at least one follow-up examination. The response rate among survivors was above 70% for all follow-ups, except at age 88, where the response rate among survivors was 59%. A flow chart of the sample is found in Figure 2.

### 3.3. Characteristics at age 70 in relation to attrition

Associations are only reported in the text if observed at more than one follow-up. Results for each follow-up examination are given in the tables.

First, we examined characteristics at age 70 in relation to refusal at each follow-up. In both unadjusted (Supplementary Table S1) and adjusted analyses (Table 3), lower educational level was associated with refusal.

**Table 3:** Characteristics at age 70 associated with refusal, death, and total attrition during follow-up.

	Refusals compared to participants				Deceased compared to participants				Total attrition compared to participants			
Characteristics at age 70 (unit)	**Follow-up at age 75**	**Follow-up at age 79**	**Follow-up at age 85**	**Follow-up at age 88**	**Follow-up at age 75**	**Follow-up at age 79**	**Follow-up at age 85**	**Follow-up at age 88**	**Follow-up at age 75**	**Follow-up at age 79**	**Follow-up at age 85**	**Follow-up at age 88**
	OR (95% CI)	OR (95% CI)	OR (95% CI)	OR (95% CI)	OR (95% CI)	OR (95% CI)	OR (95% CI)	OR (95% CI)	OR (95% CI)	OR (95% CI)	OR (95% CI)	OR (95% CI)
Female	0.7 (0.4–1.2)	1.0 (0.6–1.6)	1.0 (0.5–1.8)	1.1 (0.6–2.0)	0.3 (0.1–0.8)**	0.7 (0.4–1.0)˙	0.6 (0.4–0.99)*	0.6 (0.3–0.9)**	0.6 (0.4–0.95)*	0.8 (0.5–1.3)	0.7 (0.4–0.99)*	0.7 (0.4–1.2)
Having partner	1.0 (0.6–1.9)	1.5 (0.8–2.6)	1.5 (0.7–3.0)	1.1 (0.5–2.1)	0.4 (0.1–1.1)˙	0.8 (0.4–1.4)	0.7 (0.4–1.2)	0.7 (0.4–1.2)	0.7 (0.4–1.3)	1.0 (0.6–1.6)	0.9 (0.5–1.4)	0.7 (0.4–1.2)
More than mandatory education	0.6 (0.3–1.1)˙	**0.4 (0.2–0.7)** ***	**0.4 (0.2–0.8)** **	**0.4 (0.6–2.0)** **	**0.3 (0.1–0.8)** *	**0.5 (0.3–0.9)** *	**0.6 (0.4–0.99)** **	**0.6 (0.3–0.9)** *	**0.5 (0.3–0.9)** *	**0.5 (0.3–0.7)** ***	**0.6 (0.3–0.9)** **	**0.5 (0.3–0.8)** **
Current smoker	1.6 (0.8–2.9)	1.4 (0.7–2.7)	1.9 (0.8–4.2)	0.7 (0.2–2.0)	1.4 (0.4–4.6)	2.8 (1.5–5.1)***	2.3 (1.2–4.5)*	**3.3 (1.8–5.9)** ***	1.5 (0.9–2.7)	2.1 (1.2–3.5)**	2.7 (1.5–4.8)***	1.9 (0.9–3.6)˙
Alcohol risk consumption	0.9 (0.4–1.9)	1.0 (0.4–2.0)	0.9 (0.3–2.1)	1.2 (0.5–3.0)	1.6 (0.5–5.0)	1.1 (0.5–2.1)	0.8 (0.4–1.5)	1.0 (0.5–1.9)	1.1 (0.6–2.1)	1.0 (0.6–1.8)	0.9 (0.4–1.5)	1.0 (0.5–2.0)
ADL dependent	1.0 (0.4–2.3)	0.9 (0.3–2.2)	1.4 (0.4–3.9)	2.5 (0.8–7.7)˙	2.9 (0.9–8.9)˙	2.6 (1.3–5.2)**	3.0 (1.2–7.4)*	**2.7 (1.3–5.4)** **	1.4 (0.7–2.6)	1.7 (0.9–3.0)˙	2.4 (1.2–4.6)*	2.8 (1.1–6.8)*
Myocardial infarction	0.8 (0.3–1.8)	1.0 (0.4–2.4)	0.2 (0.0–1.7)	1.2 (0.3–3.9)	2.1 (0.7–6.4)	2.3 (1.1–4.5)*	2.7 (1.2–6.0)*	**3.0 (1.5–5.6)** ***	1.0 (0.5–2.0)	1.6 (0.9–2.9)˙	2.2 (1.1–4.2)*	2.3 (1.04–5.1)*
Atrial fibrillation	1.3 (0.6–2.9)	1.6 (0.7–3.5)	**2.9 (1.1–7.4)** *	1.4 (0.4–4.0)	**4.0 (1.4–11.2)** **	**2.4 (1.2–4.7)** **	2.0 (0.9–4.4)˙	**2.6 (1.3–5.2)** **	1.7 (0.9–3.1)˙	**1.9 (1.07–3.5)** **	**2.5 (1.3–5.0)** **	1.8 (0.8–4.0)
Heart failure	1.5 (0.4–5.1)	2.0 (0.5–7.4)	1.4 (0.2–8.5)	2.1 (0.1–24.5)	2.4 (0.4–12.6)	**3.3 (1.07–10.4)** *	5.9 (0.7–45.3)˙	**3.6 (1.005–13.2)** *	2.0 (0.7–5.4)	2.7 (0.9–7.6)˙	3.1 (0.8–11.0)˙	4.9 (0.6–37.7)
Treatment for diabetes	1.0 (0.4–2.4)	1.5 (0.6–3.4)	0.4 (0.0–2.0)	0.3 (0.0–2.7)	1.8 (0.4–6.5)	2.0 (0.9–4.4)˙	**3.4 (1.2–9.0)** *	**2.4 (1.1–4.9)** *	1.2 (0.5–2.4)	1.8 (0.9–3.5)˙	1.8 (0.8–3.7)	2.6 (0.9–6.8)˙
Treatment for hypertension	1.1 (0.6–1.8)	1.0 (0.6–1.8)	1.2 (0.6–2.2)	2.3 (1.1–4.2)*	0.3 (0.0–1.4)	1.0 (0.5–1.7)	**2.1 (1.2–3.5)** **	1.2 (0.7–1.9)	0.9 (0.5–1.5)	1.0 (0.6–1.6)	1.2 (0.7–1.8)	**2.0 (1.2–3.4)** **
Stroke	1.6 (0.6–4.4)	0.8 (0.2–2.5)	2.9 (0.6–12.2)	2.1 (0.1–24.5)	2.1 (0.4–10.4)	1.0 (0.3–3.0)	11.3 (1.4–85.2)*	**4.8 (1.5–14.8)** **	1.5 (0.6–3.6)	0.9 (0.3–2.1)	**4.1 (1.3–12.3)** *	**8.8 (1.1–66.5)** *
Dementia	1.4 (0.3–5.3)	2.0 (0.4–9.4)	2.1 (0.1–34.9)	1.1 (0.0–18.4)	1.5 (0.1–13.3)	**5.1 (1.3–18.9)** *	5.0 (0.6–39.7)	**12.0 (1.5–93.9)** *	1.6 (0.5–5.1)	**3.8 (1.2–12.5)** *	**9.2 (1.1–71.2)** *	4.0 (0.5–31.6)
APOE e4	1.0 (0.6–1.8)	1.2 (0.7–2.1)	0.8 (0.4–1.6)	1.1 (0.5–2.2)	1.1 (0.3–3.0)	1.3 (0.7–2.2)	1.2 (0.7–2.0)	1.1 (0.6–1.7)	1.0 (0.6–1.6)	1.2 (0.8–1.9)	1.1 (0.7–1.6)	1.2 (0.7–1.9)
BMI (per 5 kg/m^2^ increase)	1.1 (0.8–1.5)	0.8 (0.6–1.2)	1.0 (0.6–1.4)	0.7 (0.5–1.1)	0.5 (0.2–1.1)˙	1.1 (0.8–1.5)	1.0 (0.7–1.3)	0.9 (0.6–1.2)	1.0 (0.7–1.3)	1.0 (0.7–1.3)	1.0 (0.8–1.3)	0.9 (0.6–1.2)
SBP (per 10 mmHg increase)	1.0 (0.9–1.2)	1.0 (0.9–1.2)	1.1 (0.9–1.3)	1.1 (0.9–1.3)	1.0 (0.7–1.2)	0.9 (0.8–1.1)˙	1.0 (0.8–1.1)	1.0 (0.9–1.1)	1.0 (0.9–1.2)	1.0 (0.8–1.1)	1.0 (0.8–1.1)	1.0 (0.9–1.2)
DBP (per 10 mmHg increase)	1.1 (0.8–1.4)	1.0 (0.7–1.3)	1.2 (0.9–1.6)	**1.4 (1.04–1.9)** *	1.0 (0.6–1.6)	0.9 (0.6–1.1)	0.9 (0.7–1.2)	1.0 (0.9–1.1)	1.1 (0.8–1.3)	0.9 (0.7–1.2)	1.0 (0.8–1.2)	1.1 (0.9–1.4)
PEF (per 10% increase)	0.9 (0.8–1.1)	0.9 (0.8–1.1)˙	1.0 (0.8–1.1)	1.0 (0.8–1.2)	0.9 (0.7–1.1)	**0.8 (0.7–0.91)** ***	**0.8 (0.7–0.92)** ***	**0.9 (0.8–0.97)** **	0.9 (0.8–1.1)	**0.9 (0.7–0.94)** ***	**0.9 (0.8–0.95)** **	**0.9 (0.8–0.98)** *
Gait speed (per 1 m/s increase)	0.5 (0.1–1.9)	0.6 (0.1–2.5)	0.6 (0.1–2.8)	3.4 (0.6–18.1)	0.1 (0.0–0.7)*	**0.1 (0.0–0.4)** ***	**0.3 (0.0–0.99)** *	**0.2 (0.0–0.5)** ***	**0.3 (0.1–0.95)** *	**0.2 (0.0–0.6)** **	**0.2 (0.0–0.6)** **	0.5 (0.1–1.6)
Word fluency (per 5 words increase)	0.9 (0.7–1.2)	1.0 (0.8–1.2)	0.9 (0.7–1.2	1.0 (0.7–1.2)	0.9 (0.6–1.4)	0.7 (0.5–0.9)**	**0.7 (0.6–0.9)** ***	**0.8 (0.6–0.93)** **	1.0 (0.8–1.2)	**0.8 (0.7–0.96)** **	**0.8 (0.6–0.9)** ***	**0.8 (0.7–0.97)** *
Free recall (per 1 object increase)	0.9 (0.8–1.1)	0.9 (0.7–1.1)	0.9 (0.7–1.1)	0.9 (0.7–1.1)	0.8 (0.6–1.1)	0.8 (0.7–0.97)*	**0.9 (0.7–0.99)** *	**0.9 (0.7–0.97)** **	0.9 (0.8–1.2)˙	**0.9 (0.7–0.96)** **	**0.9 (0.7–0.994)** **	**0.9 (0.7–0.994)** *
Medications (per 1 medication increase)	**1.1 (0.01–0.2)** *	1.1 (0.9–1.2)	1.0 (0.9–1.2)	1.0 (0.8–1.1)	1.2 (1.0–1.4)˙	1.2 (1.09–1.3)***	**1.1 (1.04–1.3)** **	**1.1 (1.05–1.3)** ***	**1.1 (1.0–1.2)** *	**1.1 (1.04–1.2)** **	**1.1 (1.02–1.2)** *	**1.1 (1.008–1.2)** *
MADRS (per 5 points increase)	1.1 (0.8–1.4)	0.9 (0.7–1.2)	0.9 (0.6–1.3)	1.0 (0.7–1.3)	1.6 (1.1–2.3)**	1.3 (1.05–1.6)*	1.2 (0.9–1.5)˙	1.1 (0.9–1.5)	1.2 (0.9–1.5)˙	1.1 (0.9–1.3)	1.1 (0.9–1.4)	1.1 (0.8–1.4)

ADL, Activities of Daily Living; APOE, Apolipoprotein E; BMI, Body Mass Index; SBP, Systolic Blood Pressure; DBP, Diastolic Blood Pressure; PEF, Peak Expiratory Flow; MADRS, Montgomery Åsberg Depression Rating Scale.

Statistical analyses: logistic regression, adjusted for sex and education.

˙ *p* < 0.1.

* *p* < 0.05.

** *p* < 0.01.

*** *p* < 0.001; bolded numbers are significant at *p* < 0.05.

Second, we examined characteristics at age 70 in relation to attrition due to death before each follow-up. In both unadjusted (Supplementary Table S2) and adjusted (Table 3) analyses, male sex, lower educational level, smoking, ADL dependency, myocardial infarction, atrial fibrillation, heart failure, diabetes, stroke, dementia, lower PEF, slower gait speed, lower scores on cognitive tests, larger number of medications, and higher MADRS scores were associated with attrition due to death.

Third, we examined characteristics at age 70 in relation to total attrition before each follow-up. In both unadjusted (Supplementary Table S3) and adjusted analyses (Table 3), lower educational level, smoking, ADL dependency, stroke, dementia, lower PEF, slower gait speed, lower scores on cognitive tests, and larger number of medications were associated with total attrition. In addition, heart failure was associated with total attrition in the unadjusted analyses and male sex, myocardial infarction, and atrial fibrillation were associated with total attrition in the adjusted analyses.

All results including scores on cognitive tests remained when excluding individuals with baseline dementia.

### 3.4. Characteristics at age 75 in relation to attrition

First, we examined characteristics at age 75 in relation to refusal at each follow-up. In both unadjusted (Supplementary Table S4) and adjusted analyses (Table 4), lower educational level, higher blood pressure, and lower scores on cognitive tests were associated with refusal.

**Table 4:** Characteristics at age 75 associated with refusal, death, and total attrition during follow-up.

	Refusals compared to participants			Deceased compared to participants			Total attrition compared to participants		
Characteristics at age 75 (unit)	**Follow-up at age 79**	**Follow-up at age 85**	**Follow-up at age 88**	**Follow-up at age 79**	**Follow-up at age 85**	**Follow-up at age 88**	**Follow-up at age 79**	**Follow-up at age 85**	**Follow-up at age 88**
	OR (95% CI)	OR (95% CI)	OR (95% CI)	OR (95% CI)	OR (95% CI)	OR (95% CI)	OR (95% CI)	OR (95% CI)	OR (95% CI)
Females	1.2 (0.7–1.8)	1.1 (0.7–1.8)	0.8 (0.5–1.3)	**0.6 (0.4–0.99)** *	**0.6 (0.4–0.8)** ***	**0.6 (0.4–0.9)** **	0.8 (0.6–1.2)	**0.7 (0.5–0.94)** *	**0.7 (0.4–0.92)** *
Having partner	1.3 (0.8–2.0)	1.3 (0.7–2.2)	1.1 (0.6–1.9)	0.7 (0.4–1.1)	**0.7 (0.4–0.95)** *	**0.6 (0.4–0.9)** **	1.0 (0.6–1.4)	0.8 (0.5–1.1)	0.7 (0.5–1.1)˙
More than mandatory education	**0.5 (0.3–0.8)** ***	**0.5 (0.3–0.8)** **	**0.7 (0.4–1.1)** **	**0.6 (0.3–0.9)** **	**0.6 (0.4–0.99)** **	**0.7 (0.4–0.92)** *	**0.5 (0.3–0.7)** ***	**0.6 (0.5–0.94)** ***	**0.6 (0.4–0.9)** **
Current smoker	1.3 (0.7–2.4)	2.0 (0.9–4.2)˙	1.2 (0.4–2.8)	**3.5 (1.9–6.1)** ***	**3.8 (2.2–6.4)** ***	**3.4 (1.8–6.3)** ***	2.0 (1.2–3.1)**	**3.2 (1.9–5.3)** ***	**2.7 (1.4–5.0)** **
Alcohol risk consumption	0.8 (0.4–1.5)	0.9 (0.4–1.7)	0.8 (0.4–1.5)	1.2 (0.6–2.3)	0.9 (0.5–1.4)	0.7 (0.4–1.1)˙	0.9 (0.5–1.4)	0.9 (0.5–1.3)	0.7 (0.4–1.1)˙
ADL dependent	0.9 (0.4–1.9)	0.8 (0.3–2.0)	0.4 (0.1–1.3)	**4.9 (2.7–9.0)** ***	**4.9 (2.9–8.3)** ***	**3.9 (2.1–7.1)** ***	2.2 (1.4–3.6) ***	**3.3 (2.0–5.5)** ***	**2.6 (1.4–4.7)** **
Myocardial infarction	1.5 (0.8–2.6)	1.8 (0.9–3.6)˙	1.1 (0.5–2.5)	**2.2 (1.2–3.9)** **	**2.4 (1.4–3.9)** ***	**2.4 (1.3–4.4)** **	**1.8 (1.1–2.9)** **	**2.1 (1.3–3.4)** **	**2.1 (1.1–3.8)** *
Atrial fibrillation	1.1 (0.5–2.1)	1.6 (0.8–3.3)	0.8 (0.3–1.9)	1.3 (0.7–2.5)	1.6 (0.9–2.8)˙	1.4 (0.8–2.5)	1.2 (0.7–1.9)	1.6 (0.9–2.6)˙	1.3 (0.7–2.2)
Heart failure	1.7 (0.6–4.4)	1.2 (0.3–4.9)	1.5 (0.3–6.2)	**3.1 (1.2–7.3)** *	**4.0 (1.6–9.5)** **	**3.3 (1.1–9.8)** *	**2.6 (1.3–5.3)** **	**3.2 (1.3–7.6)** **	2.8 (0.9–8.2)˙
Treatment for diabetes	0.9 (0.4–1.8)	1.0 (0.4–2.4)	1.1 (0.4–2.7)	**2.3 (1.3–4.1)** **	**3.1 (1.8–5.2)** ***	**3.0 (1.6–5.7)** ***	1.6 (0.9–2.5)˙	**2.4 (1.4–4.0)** ***	**2.4 (1.3–4.5)** **
Treatment for hypertension	1.0 (0.6–1.6)	1.4 (0.9–2.3)	**1.6 (1.005–2.6)** *	0.9 (0.5–1.6)	**1.5 (1.0–2.1)** *	**1.7 (1.2–2.5)** **	1.0 (0.7–1.4)	**1.4 (1.05–2.0)** *	**1.7 (1.1–2.4)** **
Stroke	0.5 (0.2–1.3)	**2.3 (1.01–5.1)** *	1.3 (0.4–3.6)	**2.2 (1.2–4.1)** **	**3.7 (2.0–6.7)** ***	**3.8 (1.8–7.9)** ***	1.2 (0.7–2.1)	**3.2 (1.8–5.7)** ***	**3.0 (1.4–6.3)** **
Dementia	2.2 (0.8–5.4)˙	2.1 (0.5–7.7)	1.5 (0.2–11.3)	**8.7 (4.2–17.9)** ***	**7.0 (2.8–17.1)** ***	**11.2 (2.6–46.8)** ***	**4.4 (2.3–8.5)** ***	**5.6 (2.3–13.6)** ***	**8.4 (2.0–35.3)** **
APOE e4	1.4 (0.9–2.2)	1.0 (0.5–1.7)	1.1 (0.6–1.9)	1.1 (0.6–1.9)	1.2 (0.8–1.8)	1.3 (0.8–1.9)	1.2 (0.8–1.7)	1.2 (0.8–1.7)	1.2 (0.8–1.8)
BMI (per 5 kg/m^2^ increase)	0.8 (0.6–1.1)	1.1 (0.8–1.5)	0.9 (0.6–1.3)	0.9 (0.7–1.3)	1.0 (0.8–1.3)	1.0 (0.8–1.3)	0.9 (0.7–1.2)	1.0 (0.8–1.3)	1.0 (0.8–1.3)
SBP (per 10 mmHg increase)	**1.1 (1.03–1.3)** **	**1.2 (1.04–1.3)** **	**1.2 (1.1–1.4)** ***	**0.9 (0.7–0.98)** *	1.0 (0.9–1.1)	**1.1 (1.008–1.2)** *	1.0 (0.9–1.1)	1.0 (0.9–1.2)	**1.1 (1.03–1.3)** **
DBP (per 10 mmHg increase)	**1.3 (1.05–1.6)** *	**1.3 (1.004–1.6)** *	**1.4 (1.1–1.8)** **	0.9 (0.6–1.1)	0.9 (0.7–1.1)	1.0 (0.8–1.2)	1.1 (0.9–1.3)	1.0 (0.8–1.2)	1.1 (0.9–1.3)
PEF (per 10% increase)	1.0 (0.9–1.1)	1.0 (0.9–1.1)	1.0 (0.9–1.1)	**0.8 (0.6–0.9)** ***	**0.8 (0.7–0.9)** ***	**0.9 (0.7–0.92)** ***	**0.9 (0.8–0.95)** ***	**0.9 (0.8–0.93)** ***	**0.9 (0.8–0.96)** ***
Gait speed (per 1 m/s increase)	0.4 (0.1–1.5)	0.5 (0.1–2.1)	1.3 (0.2–5.8)	**0.1 (0.0–0.4)** **	**0.1 (0.0–0.4)** ***	**0.1 (0.0–3)** ***	**0.2 (0.0–0.7)** **	**0.2 (0.0–0.5)** ***	**0.2 (0.0–1.6)** **
Word fluency (per 5 words increase)	**0.8 (0.6–0.96)** *	**0.8 (0.6–0.93)** **	**0.8 (0.6–0.97)** *	**0.7 (0.5–0.9)** ***	**0.7 (0.5–0.8)** ***	**0.6 (0.5–0.8)** ***	**0.8 (0.6–0.9)** ***	**0.7 (0.6–0.9)** ***	**0.7 (0.5–0.8)** ***
Free recall (per 1 object increase)	**0.9 (0.7–0.97)** **	**0.8 (0.7–0.95)** **	0.9 (0.8–1.1)	**0.8 (0.6–0.9)** ***	**0.9 (0.7–0.95)** **	**0.8 (0.7–0.91)** ***	**0.8 (0.7–0.91)** ***	**0.9 (0.8–0.94)** ***	**0.9 (0.7–0.93)** ***
Medications (per 1 medication increase)	1.0 (0.9–1.1)	1.0 (0.9–1.2)	1.0 (0.9–1.2)	**1.2 (1.1–1.3)** ***	**1.2 (1.1–1.3)** ***	**1.2 (1.1–1.4)** ***	**1.1 (1.03–1.2)** ***	**1.2 (1.1–1.3)** ***	**1.2 (1.1–1.3)** ***
MADRS (per 5 points increase)	1.0 (0.8–1.2)	1.0 (0.7–1.2)	1.0 (0.8–1.2)	**1.4 (1.1–1.7)** ***	**1.4 (1.1–1.6)** ***	**1.3 (1.1–1.6)** ***	**1.2 (1.03–1.4)** *	**1.3 (1.1–1.5)** ***	**1.2 (1.07–1.5)** **

ADL, Activities of Daily Living; APOE, Apolipoprotein E; BMI, Body Mass Index; SBP, Systolic Blood Pressure; DBP, Diastolic Blood Pressure; PEF, Peak Expiratory Flow; MADRS, Montgomery Åsberg Depression Rating Scale. Statistical analyses: logistic regression, adjusted for sex and education.

˙ *p* < 0.1.

* *p* < 0.05.

** *p* < 0.01.

*** *p* < 0.001; bolded numbers are significant at *p* < 0.05.

Second, we examined characteristics at age 75 in relation to attrition due to death before each follow-up. In both unadjusted (Supplementary Table S5) and adjusted (Table 4) analyses, male sex, lower educational level, smoking, ADL dependency, myocardial infarction, heart failure, diabetes, hypertension, stroke, dementia, lower PEF, slower gait speed, lower scores on cognitive tests, larger number of medications, and higher MADRS scores were associated with attrition due to death. Lower SBP was associated with attrition due to death before age 79, while higher SBP was associated with attrition due to death before age 88. In addition, not having a partner was associated with attrition due to death in the adjusted analyses.

Third, we examined characteristics at age 75 in relation to total attrition before each follow-up. In both unadjusted (Supplementary Table S6) and adjusted (Table 4) analyses, male sex, lower educational level, smoking, ADL dependency, myocardial infarction, heart failure, diabetes, hypertension, stroke, dementia, lower PEF, slower gait speed, lower scores on cognitive tests, larger number of medications, and higher MADRS score were associated with total attrition.

All results including scores on cognitive tests remained when excluding individuals with baseline dementia.

## 4. Discussion

This paper describes longitudinal attrition in the 1930 cohort of the Gothenburg H70 Birth Cohort Studies, followed from age 70 or 75 to age 88. Lower educational and cognitive level at baseline were related to attrition due to both refusal and death. In addition, male sex, and a large number of health-related factors were related to attrition due to death. Thus, both non-death attrition and attrition due to death made the sample more selected over time, which is important to consider when evaluating results from longitudinal studies among older adults.

Among the various reasons for attrition, non-death attrition is suggested to be the most important factor in relation to representativeness, since death occurs both in the sample and in the target population (1). We found that lower cognitive and educational level were repeatedly associated with refusal at both short- and long-term follow-up. These results are in line with two previous literature reviews reporting that cognitive impairment (27, 28) and fewer years of education (27) are two of the most important factors for non-death attrition, in addition to high age (27, 28) and lower socio-economic status (27). The Medical Research Council on Cognitive Function and Ageing Study (MRC CFAS) analyzed refusals separately from other types of non-death attrition and found also that those with poorer cognitive ability and fewer years of education were more likely to refuse participation (29). This may impact longitudinal studies on disorders where lower educational and cognitive level at baseline are risk factors or determinants of the outcome, e.g., studies on dementia incidence. Other factors repeatedly studied in relation to non-death attrition are factors related to health and functional status. However, these results are more disparate (27, 28). We found no associations between refusal and indicators for poor baseline health (e.g., number of medications, gait speed, lung function, heart disease, diabetes, depressive symptoms, stroke, dementia, ADL-dependency), with the exception of blood pressure levels where those with higher blood pressure were more likely to refuse subsequent examinations. One reason why most indicators for poor health were not associated with refusal in our study may be that disorders leading to refusal might also have developed after baseline, which is especially important in studies with long follow-up and at high ages. In addition, those with disorders at baseline (when baseline was at age 75) died more often before follow-up and could therefore not refuse. Another reason may be that we offered home visits for those who were too ill to come to the outpatient department, lowering the threshold for participation. Findings regarding the influence of sex on refusal are also disparate. It has been reported that men are less likely to participate, that women are less likely to participate or, as in our study, that sex was not associated with refusal (27).

We found that a large number of social and health related factors were related to attrition due to death. In concordance with MRC CFAS (29), we found that male sex, smoking, ADL dependency, and lower cognitive level were associated with attrition due to death. In addition, we found that number of medications, heart diseases, poorer lung function, and higher MADRS score were associated with attrition due to death. However, although MRC CFAS found that poor self-perceived health was associated with attrition due to death, they did not find any associations with chronic disorders or self-reported depression. One reason for the disparate results may be the shorter follow-up time of two years in the MRC CFAS study, compared to the three to 18-year follow-up in the present study. Regarding blood pressure level, we found that lower SBP at age 75 was associated with attrition due to death at short-term follow-up, while higher SBP at age 75 was associated with attrition due to death at long-term follow-up. One reason may be that high blood pressure is a risk factor for several disorders in the longer perspective, while disorders that have already started to develop may lower blood pressure. One example is that studies on the relation between blood pressure and dementia report that blood pressure declines during the years before dementia onset (4) and is lower in those who already have started to develop the disease (30), while high blood pressure is a risk factor for dementia in the longer perspective (4).

Selective survival during follow-up may introduce bias in association studies if the exposure or outcome is related to survival (2, 31). For example, we found that both dementia and potential risk factors for dementia, such as lower educational level, smoking, and heart diseases were associated with attrition due to death, which may bias the impact of these risk factors on dementia if not accounted for. Thus, studies on risk factors for dementia are influenced by both attrition due to refusal and death.

As mentioned before, non-death attrition is most important for representativeness. If for example low educational level is less common in the sample than the target population at baseline, this selection bias will increase over time if low educational level is also associated with refusal during follow-up. However, also death affect representativeness of the sample in relation to the target population. If low educational level is associated with death, representativeness of the examined sample may actually increase over time, or at least, the increasing selection bias caused by refusal may be reduced. This is however only true if the prevalence of low education is less than half (1). If the prevalence is more than half in the examined sample and the target population, representativeness will instead decrease during follow-up (1). This illustrates the importance of describing and analysing different types of attrition separately, since they may affect representativeness in different directions.

A strength of the present study is the possibility to analyse a large number of characteristics in relation to different types of attrition (i.e., refusal, death, and total attrition) in a longitudinal population-based sample of older adults followed over 18 years. Another strength is the use of different sources of information, including interviews and physical examinations performed by health professionals, and access to high quality register data. However, there are also limitations. First, the number of some characteristics were small, and missing data was more prevalent for some characteristics (gait speed and ADL dependency), leading to lower power and increasing the risk of bias. Second, the ability to communicate in Swedish was an inclusion criterion at baseline, limiting generalisability to the total population in Gothenburg.

## 5. Conclusions

Since attrition due to death and non-death attrition may impact representativeness and study results differently it is important to consider them separately. We found that the main characteristics associated with attrition due to refusal were lower educational level, higher blood pressure, and lower scores on cognitive tests. This could be handled when planning the study by reducing barriers to participation, but also oversampling individuals more likely to drop-out such as individuals with lower educational level. One further possibility is to compensate for this afterwards by using weighted analyses. Characteristics associated with attrition due to death were male sex, lower educational and cognitive level, and several health-related factors. Therefore, when including these variables in longitudinal analyses, it is important to use models handling the competing risk of death. This is especially important when studying older adults or other groups where death rates are high.

## Data Availability

All data and analyses generated during the current study are available from the corresponding author on reasonable request.
