# Supplementary material for: Attrition in the Gothenburg H70 birth cohort studies, an 18-year follow-up of the 1930 cohort
Source: Front Epidemiol. 2023 May 9;3:1151519. doi: 10.3389/fepid.2023.1151519 (PMC10910926; doi:10.3389/fepid.2023.1151519)
Supplement: Supplementary file 1 [file Table1.docx]

# Supplementary Tables

|  | **Follow-up at age 75** | | | **Follow-up at age 79** | | | **Follow-up at age 85** | | | | **Follow-up at age 88** | | |
| --- | --- | --- | --- | --- | --- | --- | --- | --- | --- | --- | --- | --- | --- |
| **Characteristics at age 70** | **Participants** | **Refusals** | **P** | **Participants** | **Refusals** | **P** | **Participants** | **Refusals** | **P** | **Participants** | | **Refusals** | **P** |
| Females, n/N (%) | 218/387 (56.3) | 45/90 (50.0) | 0.277 | 173/315 (54.9) | 54/96 (56.3) | 0.819 | 125/213 (58.7) | 44/73 (60.3) | 0.812 | 76/129 (58.9) | | 51/82 (62.2) | 0.635 |
| Having partner, n/N (%) | 260/381 (68.2) | 61/86 (70.9) | 0.627 | 211/314 (67.2) | 69/95 (72.6) | 0.319 | 144/213 (67.6) | 54/72 (75.0) | 0.240 | 91/129 (70.5) | | 57/81 (70.4) | 0.979 |
| More than mandatory education, n/N (%) | 189/386 (49.0) | 34/89 (38.2) | 0.068 | **166/315 (52.7)** | **28/96 (29.2)** | **<0.001** | **114/213 (53.5)** | **24/73 (32.9)** | **0.003** | **74/129 (57.4)** | | **30/82 (36.6)** | **0.004** |
| Current smoker, n/N (%) | 54/379 (14.2) | 18/86 (20.9) | 0.124 | 37/310 (11.9) | 15/93 (16.1) | 0.292 | 19/211 (9.0) | 11/71 (15.5) | 0.130 | 13/126 (10.3) | | 6/81 (7.4) | 0.481 |
| Alcohol risk consumption, n/N (%) | 52/351 (14.8) | 11/74 (14.9) | 0.991 | 44/291 (15.1) | 12/83 (14.5) | 0.881 | 31/196 (15.8) | 9/64 (14.1) | 0.736 | 17/120 (14.2) | | 11/71 (15.5) | 0.802 |
| ADL dependent, n/N (%) | 36/357 (10.1) | 9/81 (11.1) | 0.784 | 26/297 (8.8) | 8/87 (9.2) | 0.899 | 13/203 (6.4) | 6/64 (9.4) | 0.423 | 6/123 (4.9) | | 9/76 (11.8) | 0.079 |
| Myocardial infarction, n/N (%) | 42/387 (10.9) | 8/90 (8.9) | 0.584 | 30/315 (9.5) | 8/96 (8.3) | 0.725 | 15/213 (7.0) | 1/73 (1.4) | 0.103 | 8/129 (6.2) | | 5/82 (6.1) | 0.976 |
| Atrial fibrillation, n/N (%) | 36/387 (9.3) | 11/90 (12.2) | 0.404 | 27/315 (8.6) | 11/96 (11.5) | 0.394 | 13/213 (6.1) | 9/73 (12.3) | 0.091 | 9/129 (7.0) | | 7/82 (8.5) | 0.677 |
| Heart failure, n/N (%) | 10/387 (2.6) | 4/90 (4.4) | 0.352 | 6/315 (1.9) | 4/96 (4.2) | 0.219 | 3/213 (1.4) | 2/73 (2.7) | 0.462 | 1/129 (0.8) | | 2/82 (2.4) | 0.346 |
| Diabetes treatment, n/N (%) | 30/387 (7.8) | 7/89 (7.9) | 0.971 | 20/315 (6.3) | 9/96 (9.4) | 0.314 | 12/213 (5.6) | 2/73 (2.7) | 0.333 | 5/129 (3.9) | | 1/82 (1.2) | 0.284 |
| Hypertension treatment, n/N (%) | 105/381 (27.6) | 25/89 (28.1) | 0.920 | 84/311 (27.0) | 27/96 (28.1) | 0.830 | 54/211 (25.6) | 21/73 (28.8) | 0.596 | **23/127 (18.1)** | | **27/82 (32.9)** | **0.015** |
| Stroke, n/N (%) | 18/387 (4.7) | 6/90 (6.7) | 0.433 | 16/315 (5.1) | 4/96 (4.2) | 0.716 | 4/213 (1.9) | 4/73 (5.5) | 0.124 | 1/129 (0.8) | | 2/82 (2.4) | 0.346 |
| Dementia, n/N (%) | 9/375 (2.4) | 3/84 (3.6) | 0.546 | 4/308 (1.3) | 3/91 (3.3) | 0.218 | 1/207 (0.5) | 1/70 (1.4) | 0.441 | 1/125 (0.8) | | 1/79 (1.3) | 0.744 |
| APOE e4, n/N (%) | 111/385 (28.8) | 23/79 (29.1) | 0.960 | 86/315 (27.3) | 28/89 (31.5) | 0.442 | 60/213 (28.2) | 17/68 (25.0) | 0.610 | 34/129 (26.4) | | 22/78 (28.2) | 0.772 |
| BMI (kg/m^2^), mean ± SD (N) | 26.9±4.2 (381) | 27.2±4.2 (85) | 0.595 | 26.9±4.2 (314) | 26.5±3.7 (95) | 0.323 | 26.9±3.8 (211) | 26.8±4.0 (73) | 0.880 | 27.3±4.1 (128) | | 26.6±3.8 (82) | 0.171 |
| SBP (mmHG), mean ± SD (N) | 155±22 (386) | 155±21 (88) | 0.840 | 156±22 (314) | 155±20 (96) | 0.848 | 156±22 (213) | 157±20 (73) | 0.562 | 154±20 (129) | | 157±22 (82) | 0.369 |
| DBP (mmHG), mean ± SD (N) | 84±11 (386) | 84±11 (88) | 0.523 | 84±11 (314) | 84±10 (96) | 0.925 | 84±11 (213) | 86±10 (73) | 0.231 | **83±10 (129)** | | **86±11 (82)** | **0.023** |
| PEF (% of expected) , mean ± SD (N) | 97±24 (365) | 93±23 (80) | 0.160 | **99±23 (301)** | **94±23 (91)** | **0.046** | 100±22 (205) | 98±23 (69) | 0.499 | 101±24 (125) | | 98±18 (80) | 0.438 |
| Gait speed (m/s), mean ± SD (N) | 1.30±0.21 (315) | 1.27±0.20 (67) | 0.207 | 1.32±0.20 (268) | 1.28±0.21 (67) | 0.237 | 1.33±0.21 (177) | 1.30±0.20 (54) | 0.316 | 1.32±0.20 (104) | | 1.35±0.20 (64) | 0.337 |
| Word fluency, mean ± SD (N) | 22.8±6.9 (370) | 22.0±6.5 (80) | 0.333 | 23.5±6.7 (303) | 22.5±7.0 (90) | 0.222 | 24.1±6.8 (205) | 22.7±6.6 (69) | 0.155 | 24.2±6.5 (124) | | 23.1±6.9 (78) | 0.263 |
| Free recall, mean ± SD (N) | 7.1±1.9 (370) | 6.8±1.8 (79) | 0.124 | 7.2±1.8 (303) | 6.9±1.8 (90) | 0.119 | 7.3±1.8 (206) | 7.1±1.6 (69) | 0.263 | 7.4±1.7 (125) | | 7.1±1.9 (78) | 0.264 |
| Medications, median [IQR] (N) | 2 [1-5], (387) | 3 [1-1.5], (89) | 0.073 | 2 [1-4], (315) | 3 [1-5], (96) | 0.242 | 2 [1-4], (213) | 3 [1-4], (73) | 0.972 | 2 [1-4], (129) | | 2 [1-4], (82) | 0.543 |
| MADRS, median [IQR] (N) | 3 [1-6], (364) | 3 [1-6], (78) | 0.971 | 3 [1-6], (299) | 3 [0.75-6-8], (90) | 0.692 | 3 [1-6], (203) | 3 [0-5.25], (70) | 0.887 | 3 [0-6], (123) | | 3 [0-6], (78) | 0.993 |

### Table S1. Baseline characteristics at age 70 associated with participation and refusal during follow-up, unadjusted analyses

P-values are based on logistic regression, bolded numbers are significant at p<0.05; BMI=Body Mass Index, SBP=Systolic Blood Pressure, DBP=Diastolic Blood Pressure, PEF= Peak Expiratory Flow, ADL= Activities of Daily Living, MADRS= Montgomery Åsberg Depression Rating Scale, APOE= Apolipoprotein E

### Table S2. Baseline characteristics at age 70 associated with participation and death during follow-up, unadjusted analyses

|  | **Follow-up at age 75** | | | **Follow-up at age 79** | | | **Follow-up at age 85** | | | **Follow-up at age 88** | | |
| --- | --- | --- | --- | --- | --- | --- | --- | --- | --- | --- | --- | --- |
| **Characteristics at age 70** | **Participants** | **Deceased** | **p** | **Participants** | **Deceased** | **P** | **Participants** | **Deceased** | **P** | **Participants** | **Deceased** | **P** |
| Females, n/N (%) | **218/387 (56.3)** | **7/24 (29.2)** | **0.013** | 173/315 (54.9) | 45/99 (45.5) | 0.101 | **125/213 (58.7)** | **104/227 (45.8)** | **0.007** | 76/129 (58.9) | 147/301 (48.8) | 0.056 |
| Partner, n/N (%) | 260/381 (68.2) | 12/22 (54.5) | 0.188 | 211/314 (67.2) | 60/92 (65.2) | 0.723 | 144/213 (67.6) | 142/218 (65.1) | 0.588 | 91/129 (70.5) | 192/290 (66.2) | 0.382 |
| More than mandatory education, n/N (%) | **189/386 (49.0)** | **5/22 (22.7)** | **0.023** | **166/315 (52.7)** | **36/95 (37.9)** | **0.012** | **114/213 (53.5)** | **94/223 (42.2)** | **0.018** | **74/129 (57.4)** | **128/295 (43.4)** | **0.008** |
| Current smoker, n/N (%) | 54/379 (14.2) | 4/23 (17.4) | 0.678 | **37/310 (11.9)** | **25/95 (26.3)** | **<0.001** | **19/211 (9.0)** | **50/220 (22.7)** | **<0.001** | **13/126 (10.3)** | **59/294 (20.1)** | **0.017** |
| Alcohol risk consumption, n/N (%) | 52/351 (14.8) | 6/20 (30.0) | 0.077 | 44/291 (15.1) | 15/82 (18.3) | 0.487 | 31/196 (15.8) | 31/198 (15.7) | 0.965 | 17/120 (14.2) | 42/264 (15.9) | 0.661 |
| ADL dependent, n/n (%) | **36/357 (10.1)** | **6/22 (27.3)** | **0.018** | **26/297 (8.8)** | **20/85 (23.5)** | **<0.001** | **13/203 (6.4)** | **34/202 (16.8)** | **0.002** | **6/123 (4.9)** | **39/268 (14.6)** | **0.008** |
| Myocardial infarction, n/N (%) | 42/387 (10.9) | 5/24 (20.8) | 0.145 | **30/315 (9.5)** | **18/99 (18.2)** | **0.021** | **15/213 (7.0)** | **41/227 (18.1)** | **<0.001** | **8/129 (6.2)** | **44/301 (14.6)** | **0.017** |
| Atrial fibrillation, n/N (%) | **36/387 (9.3)** | **7/24 (29.2)** | **0.004** | **27/315 (8.6)** | **18/99 (18.2)** | **0.009** | **13/213 (6.1)** | **34/227 (15.0)** | **0.003** | 9/129 (7.0) | 40/301 (13.3) | 0.063 |
| Heart failure, n/N (%) | 10/387 (2.6) | 2/24 (8.3) | 0.126 | **6/315 (1.9)** | **7/99 (7.1)** | **0.016** | **3/213 (1.4)** | **13/227 (5.7)** | **0.025** | 1/129 (0.8) | 15/301 (5.0) | 0.067 |
| Diabetes treatment, n/N (%) | 30/387 (7.8) | 3/23 (13.0) | 0.371 | 20/315 (6.3) | 12/97 (12.4) | 0.057 | **12/213 (5.6)** | **28/225 (12.4)** | **0.016** | **5/129 (3.9)** | **36/299 (12.0)** | **0.013** |
| Hypertension treatment, n/N (%) | 105/381 (27.6) | 2/23 (8.7) | 0.064 | 84/311 (27.0) | 24/95 (25.3) | 0.736 | 54/211 (25.6) | 61/221 (27.6) | 0.637 | **23/127 (18.1)** | **86/295 (29.2)** | **0.019** |
| Stroke, n/N (%) | 18/387 (4.7) | 3/24 (12.5) | 0.105 | 16/315 (5.1) | 6/99 (6.1) | 0.705 | **4/213 (1.9)** | **18/227 (7.9)** | **0.007** | **1/129 (0.8)** | **24/301 (8.0)** | **0.019** |
| Dementia, n/N (%) | 9/375 (2.4) | 2/24 (8.3) | 0.107 | **4/308 (1.3)** | **7/95 (7.4)** | **0.005** | **1/207 (0.5)** | **13/219 (5.9)** | **0.014** | **1/125 (0.8)** | 13/289 (4.5) | 0.091 |
| APOE e4, n/N (%) | 111/385 (28.8) | 7/21 (33.3) | 0.659 | 86/315 (27.3) | 29/89 (32.6) | 0.330 | 60/213 (28.2) | 65/214 (30.4) | 0.617 | 34/129 (26.4) | 88/288 (30.6) | 0.384 |
| BMI (kg/m^2^), mean ± SD (N) | 26.9±4.2 (381) | 25.7±3.1 (23) | 0.156 | 26.9±4.2 (314) | 27.3±4.3 (91) | 0.416 | 26.9±3.8 (211) | 27.0±4.4 (216) | 0.697 | 27.3±4.1 (128) | 27.0±4.4 (289) | 0.419 |
| SBP (mmHG), mean ± SD (N) | 155±22 (386) | 153±25 (23) | 0.680 | 156±22 (314) | 151±23 (96) | 0.060 | 156±22 (213) | 153±23 (223) | 0.261 | 154±20 (129) | 154±23 (297) | 0.998 |
| DBP (mmHG), mean ± SD (N) | 84±11 (386) | 84±11 (23) | 0.960 | 84±11 (314) | 82±11 (96) | 0.178 | 84±11 (213) | 83±11 (223) | 0.572 | 83±10 (129) | 84±11 (297) | 0.472 |
| PEF (% of expected) , mean ± SD (N) | 97±24 (365) | 88±27 (22) | 0.072 | **99±23 (301)** | **87±25 (87)** | **<0.001** | **100±22 (205)** | **91±25 (207)** | **<0.001** | **101±24 (125)** | **93±25 (273)** | **0.003** |
| Gait speed (m/s), mean ± SD (N) | **1.30±0.21 (315)** | **1.18±0.30 (19)** | **0.017** | **1.32±0.20 (268)** | **1.21±0.27 (71)** | **<0.001** | **1.33±0.21 (177)** | **1.25±0.22 (177)** | **<0.001** | **1.32±0.20 (104)** | **1.27±0.22 (237)** | **0.047** |
| Word fluency, mean ± SD (N) | 22.8±6.9 (370) | 21.1±7.0 (23) | 0.248 | **23.5±6.7 (303)** | **20.3±6.7 (90)** | **<0.001** | **24.1±6.8 (205)** | **21.2±6.7 (211)** | **<0.001** | **24.2±7.0 (124)** | **21.8±6.8 (280)** | **0.001** |
| Free recall, mean ± SD (N) | **7.1±1.9 (370)** | **6.2±2.2 (22)** | **0.035** | **7.2±1.8 (303)** | **6.5±2.2 (88)** | **0.002** | **7.3±1.8 (206)** | **6.7±2.1 (208)** | **<0.001** | **7.4±1.7 (125)** | **6.8±2.0 (277)** | **0.006** |
| Medications, median [IQR] (N) | 2 [1-5], (387) | 4 [1-5], (23) | 0.284 | **2 [1-4], (315)** | **3 [2-6], (97)** | **<0.001** | **2 [1-4], (213)** | **3 [1-6], (225)** | **0.008** | **2 [1-4], (129)** | **3 [1-5], (299)** | **0.016** |
| MADRS, median [IQR] (N) | **3 [1-6], (364)** | **6 [1-11.75], (22)** | **0.010** | **3 [1-6], (299)** | **3 [1-7], (87)** | **0.033** | **3 [1-6], (203)** | 3 [1-6.5], (205) | 0.249 | 3 [0-6], (123) | 3 [1-6], (273) | 0.432 |

P-values are based on logistic regression, bolded numbers are significant at p<0.05; BMI=Body Mass Index, SBP=Systolic Blood Pressure, DBP=Diastolic Blood Pressure, PEF= Peak Expiratory Flow, ADL= Activities of Daily Living, MADRS= Montgomery Åsberg Depression Rating Scale, APOE= Apolipoprotein E

### Table S3. Baseline characteristics at age 70 associated with participation and total attrition during follow-up, unadjusted analyses

|  | **Follow-up at age 75** | | | **Follow-up at age 79** | | | **Follow-up at age 85** | | | **Follow-up at age 88** | | |
| --- | --- | --- | --- | --- | --- | --- | --- | --- | --- | --- | --- | --- |
| **Characteristics at age 70** | **Participants** | **Drop-outs^a^** | **P** | **Participants** | **Drop-outs^a^** | **P** | **Participants** | **Drop-outs^a^** | **P** | **Participants** | **Drop-outs^a^** | **P** |
| Females, n/N (%) | **218/387 (56.3)** | **63/137 (46.0)** | **0.037** | 173/315 (54.9) | 108/209 (51.7) | 0.466 | 125/213 (58.7) | 156/311 (50.2) | 0.055 | 76/129 (58.9) | 205/395 (51.9) | 0.165 |
| Partner, n/N (%) | 260/381 (68.2) | 85/129 (65.9) | 0.622 | 211/314 (67.2) | 134/196 (68.4) | 0.784 | 144/213 (67.6) | 201/297 (67.7) | 0.986 | 91/129 (70.5) | 254/381 (66.7) | 0.416 |
| More than mandatory education, n/N (%) | **189/386 (49.0)** | **45/131 (34.4)** | **0.004** | **166/315 (52.7)** | **68/202 (33.7)** | **<0.001** | **114/213 (53.5)** | **120/304 (39.5)** | **0.002** | **74/129 (57.4)** | **160/388 (41.2)** | **0.002** |
| Current smoker, n/N (%) | 54/379 (14.2) | 26/131 (19.8) | 0.130 | **37/310 (11.9)** | **43/200 (21.5)** | **0.004** | **19/211 (9.0)** | **61/299 (20.4)** | **<0.001** | 13/126 (10.3) | 67/384 (17.4) | 0.059 |
| Alcohol risk consumption, n/N (%) | 52/351 (14.8) | 20/110 (18.2) | 0.397 | 44/291 (15.1) | 28/170 (16.5) | 0.700 | 31/196 (15.8) | 41/265 (15.5) | 0.920 | 17/120 (14.2) | 55/341 (16.1) | 0.611 |
| ADL dependent, n/n (%) | 36/357 (10.1) | 19/120 (15.8) | 0.091 | **26/297 (8.8)** | **29/180 (16.1)** | **0.016** | **13/203 (6.4)** | **42/274 (15.3)** | **0.003** | **6/123 (4.9)** | **49/354 (13.8)** | **0.010** |
| Myocardial infarction, n/N (%) | 42/387 (10.9) | 15/137 (10.9) | 0.975 | 30/315 (9.5) | 27/209 (12.9) | 0.223 | **15/213 (7.0)** | **42/311 (13.5)** | **0.022** | 8/129 (6.2) | 49/395 (12.4) | 0.054 |
| Atrial fibrillation, n/N (%) | 36/387 (9.3) | 20/137 (14.6) | 0.087 | 27/315 (8.6) | 29/209 (13.9) | 0.056 | **13/213 (6.1)** | **43/311 (13.8)** | **0.006** | 9/129 (7.0) | 47/395 (11.9) | 0.121 |
| Heart failure, n/N (%) | 10/387 (2.6) | 8/137 (5.8) | 0.080 | **6/315 (1.9)** | **12/209 (7.1)** | **0.024** | **3/213 (1.4)** | **15/311 (4.8)** | **0.047** | 1/129 (0.8) | 17/395 (4.3) | 0.091 |
| Diabetes treatment, n/N (%) | 30/387 (7.8) | 12/135 (8.9) | 0.676 | 20/315 (6.3) | 22/207 (10.6) | 0.082 | 12/213 (5.6) | 30/309 (9.7) | 0.096 | 5/129 (3.9) | 37/393 (9.4) | 0.052 |
| Hypertension treatment, n/N (%) | 105/381 (27.6) | 33/133 (24.8) | 0.538 | 84/311 (27.0) | 54/203 (26.6) | 0.919 | 54/211 (25.6) | 84/303 (27.7) | 0.592 | **23/127 (18.1)** | **115/387 (29.7)** | **0.011** |
| Stroke, n/N (%) | 18/387 (4.7) | 9/137 (6.6) | 0.385 | 16/315 (5.1) | 11/209 (5.3) | 0.929 | **4/213 (1.9)** | **23/311 (7.4)** | **0.009** | **1/129 (0.8)** | **26/395 (6.6)** | **0.032** |
| Dementia, n/N (%) | 9/375 (2.4) | 6/124 (4.8) | 0.177 | **4/308 (1.3)** | **11/191 (5.8)** | **0.009** | **1/207 (0.5)** | **14/292 (4.8)** | **0.024** | 1/125 (0.8) | 14/374 (3.7) | 0.130 |
| APOE e4, n/N (%) | 111/385 (28.8) | 35/121 (28.9) | 0.984 | 86/315 (27.3) | 60/191 (31.4) | 0.323 | 60/213 (28.2) | 86/293 (29.4) | 0.772 | 34/129 (26.4) | 112/377 (29.7) | 0.469 |
| BMI (kg/m^2^), mean ± SD (N) | 26.9±4.2 (381) | 27.0±4.3 (129) | 0.823 | 26.9±4.2 (314) | 27.0±4.2 (196) | 0.812 | 26.9±3.8 (211) | 27.0±4.4 (298) | 0.715 | 27.3±4.1 (128) | 26.9±4.2 (382) | 0.270 |
| SBP (mmHG), mean ± SD (N) | 155±22 (386) | 155±22 (134) | 0.941 | 156±22 (314) | 153±21 (206) | 0.201 | 156±22 (213) | 154±22 (307) | 0.422 | 154±20 (129) | 155±22 (391) | 0.852 |
| DBP (mmHG), mean ± SD (N) | 84±11 (386) | 84±11 (134) | 0.534 | 84±11 (314) | 83±11 (206) | 0.366 | 84±11 (213) | 84±11 (307) | 0.989 | 83±10 (129) | 84±11 (391) | 0.246 |
| PEF (% of expected) , mean ± SD (N) | 97±24 (365) | 92±25 (123) | 0.060 | **99±23 (301)** | **90±24 (187)** | **<0.001** | **100±22 (205)** | **92±25 (283)** | **<0.001** | **101±24 (125)** | **94±24 (363)** | **0.007** |
| Gait speed (m/s), mean ± SD (N) | **1.30±0.21 (315)** | **1.25±0.23 (96)** | **0.025** | **1.32±0.20 (268)** | **1.24±0.24 (143)** | **<0.001** | **1.33±0.21 (177)** | **1.26±0.22 (234)** | **0.002** | 1.32±0.20 (104) | 1.28±0.22 (307) | 0.159 |
| Word fluency, mean ± SD (N) | 22.8±6.9 (370) | 22.0±6.9 (118) | 0.302 | **23.5±6.7 (303)** | **21.3±6.9 (185)** | **<0.001** | **24.1±6.8 (205)** | **21.6±6.8 (283)** | **<0.001** | **24.2±6.5 (124)** | **22.1±6.9 (364)** | **0.004** |
| Free recall, mean ± SD (N) | **7.1±1.9 (370)** | **6.7±1.9 (116)** | **0.020** | **7.2±1.8 (303)** | **6.7±2.0 (183)** | **0.001** | **7.3±1.8 (206)** | **6.8±2.0 (280)** | **0.002** | **7.4±1.7 (125)** | **6.9±2.0 (361)** | **0.012** |
| Medications, median [IQR] (N) | 2 [1-5], (387) | 3 [1-5], (135) | 0.157 | **2 [1-4], (315)** | **3 [1-5], (207)** | **0.005** | **2 [1-4], (213)** | **3 [1-5], (309)** | **0.045** | 2 [1-4], (129) | 3 [1-5], (393) | 0.083 |
| MADRS, median [IQR] (N) | 3 [1-6], (364) | 3 [1-6.75], (116) | 0.229 | 3 [1-6], (299) | 3 [1-6], (181) | 0.260 | 3 [1-6], (203) | 3 [1-6], (277) | 0.382 | 3 [0-6], (123) | 3 [1-6], (357) | 0.611 |

P-values are based on logistic regression, bolded numbers are significant at p<0.05; BMI=Body Mass Index, SBP=Systolic Blood Pressure, DBP=Diastolic Blood Pressure, PEF= Peak Expiratory Flow, ADL= Activities of Daily Living, MADRS= Montgomery Åsberg Depression Rating Scale, APOE= Apolipoprotein E
^a^ Drop-outs includes participants that have died or emigrated before follow-up, refused participation or contact failure at follow-up, or were excluded due to language difficulties or for technical reasons.

### Table S4. Characteristics at age 75 associated with participation and refusal during follow-up, unadjusted analyses

|  | **Follow-up at age 79** | | | **Follow-up at age 85** | | | **Follow-up at age 88** | | | |
| --- | --- | --- | --- | --- | --- | --- | --- | --- | --- | --- |
| **Characteristics at age 75** | **Participants** | **Refusals** | **P** | **Participants** | **Refusals** | **P** | | **Participants** | **Refusals** | **P** |
| Females, n/N (%) | 302/520 (58.1) | 81/129 (62.8) | 0.330 | 217/354 (61.3) | 71/109 (65.1) | 0.470 | | 137/215 (63.7) | 81/137 (59.1) | 0.387 |
| Partner, n/N (%) | 319/514 (62.1) | 81/127 (63.8) | 0.721 | 223/352 (63.4) | 71/108 (65.7) | 0.651 | | 140/215 (65.1) | 91/134 (67.9) | 0.592 |
| More than mandatory education, n/N (%) | **284/520 (54.6)** | **46/128 (35.9)** | **<0.001** | **200/354 (56.5)** | **43/109 (39.4)** | **0.002** | | 122/215 (56.7) | 65/137 (47.4) | 0.089 |
| Current smoker, n/N (%) | 51/515 (9.9) | 17/128 (13.3) | 0.268 | **22/349 (6.3)** | **14/109 (12.8)** | **0.030** | | 13/215 (6.0) | 10/134 (7.5) | 0.605 |
| Alcohol risk consumption, n/N (%) | 89/458 (19.4) | 15/103 (14.6) | 0.252 | 60/312 (19.2) | 15/92 (16.3) | 0.526 | | 42/193 (21.8) | 22/119 (18.5) | 0.487 |
| ADL dependent, n/n (%) | 53/490 (10.8) | 13/112 (11.6) | 0.809 | 25/338 (7.4) | 7/103 (6.8) | 0.870 | | 15/202 (7.4) | 4/132 (3.0) | 0.101 |
| Myocardial infarction, n/N (%) | 56/520 (10.8) | 19/129 (14.7) | 0.210 | 30/354 (8.5) | 15/109 (13.8) | 0.107 | | 16/215 (7.4) | 12/137 (8.8) | 0.656 |
| Atrial fibrillation, n/N (%) | 57/520 (11.0) | 16/129 (12.4) | 0.643 | 31/354 (8.8) | 14/109 (12.8) | 0.211 | | 20/215 (9.3) | 11/137 (8.0) | 0.681 |
| Heart failure, n/N (%) | 15/520 (2.9) | 7/129 (5.4) | 0.160 | 7/354 (2.0) | 3/109 (2.8) | 0.628 | | 4/215 (1.9) | 4/137 (2.9) | 0.519 |
| Diabetes treatment, n/N (%) | 53/520 (10.2) | 13/129 (10.1) | 0.969 | 25/354 (7.1) | 8/109 (7.3) | 0.922 | | 13/215 (6.0) | 9/137 (6.6) | 0.843 |
| Hypertension treatment, n/N (%) | 190/506 (37.5) | 47/125 (37.6) | 0.992 | 115/345 (33.3) | 44/107 (41.1) | 0.141 | | 63/212 (29.7) | 52/132 (39.4) | 0.065 |
| Stroke, n/N (%) | 48/520 (9.2) | 7/129 (5.4) | 0.170 | **17/354 (4.8)** | **11/109 (10.1)** | **0.048** | | 9/215 (4.2) | 8/137 (5.8) | 0.482 |
| Dementia, n/N (%) | 15/519 (2.9) | 8/128 (6.3) | 0.073 | 6/352 (1.7) | 4/109 (3.7) | 0.230 | | 2/215 (0.9) | 2/135 (1.5) | 0.640 |
| APOE e4, n/N (%) | 141/514 (27.4) | 39/112 (34.8) | 0.119 | 95/353 (26.9) | 27/102 (26.5) | 0.929 | | 55/213 (25.8) | 37/133 (27.8) | 0.682 |
| BMI (kg/m^2^), mean ± SD (N) | 26.7± 4.1 (505) | 26.3± 3.5 (123) | 0.311 | 26.5±3.7 (352) | 26.7±4.0 (107) | 0.635 | | 26.5± 3.7 (213) | 26.4± 3.7 (135) | 0.671 |
| SBP (mmHG), mean ± SD (N) | **151±21 (519)** | **156±22 (126)** | **0.011** | **150±21 (354)** | **156±21 (108)** | **0.012** | | **147±19 (215)** | **155± 20 (136)** | **<0.001** |
| DBP (mmHG), mean ± SD (N) | **81±10 (518)** | **83±11 (126)** | **0.022** | 81±10 (354) | 83±10 (108) | 0.075 | | **80±9 (215)** | **84± 11 (136)** | **0.004** |
| PEF (% of expected) , mean ± SD (N) | 111±25 (487) | 110±28 (114) | 0.849 | 113±24 (334) | 112±23 (103) | 0.846 | | 113±23 (204) | 113±24 (130) | 0.916 |
| Gait speed (m/s), mean ± SD (N) | 1.19±0.19 (435) | 1.15±0.19 (88) | 0.061 | 1.20±0.18 (302) | 1.18±0.16 (88) | 0.236 | | 1.21±0.17 (185) | 1.22±0.15 (113) | 0.748 |
| Word fluency, mean ± SD (N) | **21.5±6.4 (507)** | **19.3±6.5 (123)** | **<0.001** | **22.3±6.1 (345)** | **20.0±6.0 (108)** | **<0.001** | | **23.1± 6.0 (211)** | **21.3±5.9 (133)** | **0.009** |
| Free recall, mean ± SD (N) | **7.3±1.9 (505)** | **6.8±2.1 (122)** | **0.009** | **7.4±1.9 (344)** | **6.8±2.1 (108)** | **0.005** | | 7.6±1.9 (210) | 7.3±1.8 (133) | 0.177 |
| Medications, median [IQR], (N) | 4 [1-6], (510) | 4 [2-6], (127) | 0.945 | 3 [1-5], (349) | 4 [2-6], (105) | 0.275 | | 3 [1-5], (213) | 3 [1-5], (129) | 0.420 |
| MADRS, median [IQR], (N) | 4 [2-8], (506) | 4 [2-9], (121) | 0.504 | 4 [1-8], (344) | 4 [2-9], (107) | 0.831 | | 3 [1-7], (210) | 4 [2-8], (133) | 0.997 |

P-values are based on logistic regression, bolded numbers are significant at p<0.05; BMI=Body Mass Index, SBP=Systolic Blood Pressure, DBP=Diastolic Blood Pressure, PEF= Peak Expiratory Flow, ADL= Activities of Daily Living, MADRS= Montgomery Åsberg Depression Rating Scale, APOE= Apolipoprotein E

### Table S5. Characteristics at age 75 associated with participation and death during follow-up, unadjusted analyses

|  | **Follow-up at age 79** | | | **Follow-up at age 85** | | | **Follow-up at age 88** | | |
| --- | --- | --- | --- | --- | --- | --- | --- | --- | --- |
| **Characteristics at age 75** | **Participants** | **Deceased** | **P** | **Participants** | **Deceased** | **P** | **Participants** | **Deceased** | **P** |
| Females, n/N (%) | 302/520 (58.1) | 47/98 (48.0) | 0.065 | **217/354 (61.3)** | **142/291 (48.8)** | **0.002** | **137/215 (63.7)** | **211/402 (52.5)** | **0.008** |
| Partner, n/N (%) | 319/514 (62.1) | 51/92 (55.4) | 0.231 | 223/352 (63.4) | 162/280 (57.9) | 0.160 | 140/215 (65.1) | 225/391 (57.5) | 0.069 |
| More than mandatory education, n/N (%) | **284/520 (54.6)** | **39/96 (40.6)** | **0.012** | **200/354 (56.5)** | **132/288 (45.8)** | **0.007** | **122/215 (56.7)** | **186/398 (46.7)** | **0.018** |
| Current smoker, n/N (%) | **51/515 (9.9)** | **25/89 (28.1)** | **<0.001** | **22/349 (6.3)** | **57/281 (20.3)** | **<0.001** | **13/215 (6.0)** | **70/390 (17.9)** | **<0.001** |
| Alcohol risk consumption, n/N (%) | 89/458 (19.4) | 15/65 (23.1) | 0.492 | 60/312 (19.2) | 44/228 (19.3) | 0.984 | 42/193 (21.8) | 54/317 /17.0) | 0.186 |
| ADL dependent, n/n (%) | **53/490 (10.8)** | **26/73 (35.6)** | **<0.001** | **25/338 (7.4)** | **63/243 (25.9)** | **<0.001** | **15/202 (7.4)** | **77/347 (22.2)** | **<0.001** |
| Myocardial infarction, n/N (%) | **56/520 (10.8)** | **22/98 (22.4)** | **0.002** | **30/354 (8.5)** | **56/291 (19.2)** | **<0.001** | **16/215 (7.4)** | **70/402 (17.4)** | **<0.001** |
| Atrial fibrillation, n/N (%) | 57/520 (11.0) | 15/98 (15.3) | 0.221 | **31/354 (8.8)** | **44/291 (15.1)** | **0.013** | 20/215 (9.3) | 57/402 (14.2) | 0.083 |
| Heart failure, n/N (%) | **15/520 (2.9)** | **9/98 (9.2)** | **0.005** | **7/354 (2.0)** | **23/291 (7.9)** | **<0.001** | **4/215 (1.9)** | **26/402 (6.5)** | **0.017** |
| Diabetes treatment, n/N (%) | **53/520 (10.2)** | **22/98 (22.4)** | **<0.001** | **25/354 (7.1)** | **58/291 (19.9)** | **<0.001** | **13/215 (6.0)** | **69/402 (17.2)** | **<0.001** |
| Hypertension treatment, n/N (%) | 190/506 (37.5) | 31/86 (36.0) | 0.790 | **115/345 (33.3)** | **113/272 (41.5)** | **0.036** | **63/212 (29.7)** | **157/379 (41.4)** | **0.005** |
| Stroke, n/N (%) | **48/520 (9.2)** | **19/98 (19.4)** | **0.004** | **17/354 (4.8)** | **48/291 (16.5)** | **<0.001** | **9/215 (4.2)** | **60/402 (14.9)** | **<0.001** |
| Dementia, n/N (%) | **15/519 (2.9)** | **21/95 (22.1)** | **<0.001** | **6/352 (1.7)** | **34/288 (11.8)** | **<0.001** | **2/215 (0.9)** | **41/399 (10.3)** | **<0.001** |
| APOE e4, n/N (%) | 141/514 (27.4) | 26/87 (29.9) | 0.637 | 95/353 (26.9) | 82/265 (30.9) | 0.273 | 55/213 (25.8) | 113/373 (30.3) | 0.250 |
| BMI (kg/m^2^), mean ± SD (N) | 26.7±4.1 (505) | 26.5±5.0 (90) | 0.759 | 26.5± 3.7 (352) | 26.7±4.6 (267) | 0.579 | 26.5±3.7 (213) | 26.7±4.4 (375) | 0.654 |
| SBP (mmHG), mean ± SD (N) | **151±21 (519)** | **145±23 (98)** | **0.015** | 150±21 (354) | 150±22 (288) | 0.977 | **147±19 (215)** | **151±22 (399)** | **0.034** |
| DBP (mmHG), mean ± SD (N) | 81±10 (518) | 79±11 (98) | 0.216 | 81±10 (354) | 80±11 (287) | 0.263 | 80±9 (215) | 80±11 (398) | 0.879 |
| PEF (% of expected) , mean ± SD (N) | **111±25 (487)** | **94±27 (75)** | **<0.001** | **113±25 (334)** | **101±29 (247)** | **<0.001** | **113±23 (204)** | **103±28 (348)** | **<0.001** |
| Gait speed (m/s), mean ± SD (N) | **1.19±0.19 (435)** | **1.09±0.20 (44)** | **0.002** | **1.20±0.18 (302)** | **1.13±0.22 (180)** | **<0.001** | **1.21±0.17 (185)** | **1.13±0.21 (267)** | **<0.001** |
| Word fluency, mean ± SD (N) | **21.5±6.4 (507)** | **18.6±6.6 (79)** | **<0.001** | **22.3±6.1 (345)** | **19.0±6.9 (257)** | **<0.001** | **23.1± 6.0 (211)** | **19.2±6.7 (362)** | **<0.001** |
| Free recall, mean ± SD (N) | **7.3±1.9 (505)** | **6.3±2.2 (76)** | **<0.001** | **7.4±1.9 (344)** | **6.8±2.1 (257)** | **<0.001** | **7.6±1.9 (210)** | **6.8±2.1 (363)** | **<0.001** |
| Medications, median [IQR] (N) | **4 [1-6], (510)** | **5 [3-8.5], (93)** | **<0.001** | **3 [1-5], (349)** | **5 [2-7.25], (282)** | **<0.001** | **3 [1-5], (213)** | **5 [2-7], (393)** | **<0.001** |
| MADRS, median [IQR] (N) | **4 [2-8], (506)** | **6 [3-12], (73)** | **<0.001** | **4 [1-8], (344)** | **6 [2-11], (255)** | **<0.001** | **3 [1-7], (210)** | **6 [3-11], (360)** | **<0.001** |

P-values are based on logistic regression, bolded numbers are significant at p<0.05; BMI=Body Mass Index, SBP=Systolic Blood Pressure, DBP=Diastolic Blood Pressure, PEF= Peak Expiratory Flow, ADL= Activities of Daily Living, MADRS= Montgomery Åsberg Depression Rating Scale, APOE= Apolipoprotein E

### Table S6. Characteristics at age 75 associated with participation and total attrition during follow-up, unadjusted analyses

|  | **Follow-up at age 79** | | | **Follow-up at age 85** | | | **Follow-up at age 88** | | |
| --- | --- | --- | --- | --- | --- | --- | --- | --- | --- |
| **Characteristics at age 75** | **Participants** | **Drop-outs^a^** | **P** | **Participants** | **Drop-outs^a^** | **P** | **Participants** | **Drop-outs^a^** | **P** |
| Females, n/N (%) | 302/520 (58.1) | 136/247 (55.1) | 0.430 | **217/354 (61.3)** | **221/413 (53.5)** | **0.030** | **137/215 (63.7)** | **301/552 (54.5)** | **0.021** |
| Partner, n/N (%) | 319/514 (62.1) | 145/239 (60.7) | 0.715 | 223/352 (63.4) | 241/401 (60.1) | 0.360 | 140/215 (65.1) | 324/538 (60.2) | 0.213 |
| More than mandatory education, n/N (%) | **284/520 (54.6)** | **92/243 (37.9)** | **<0.001** | **200/354 (56.5)** | **176/409 (43.0)** | **<0.001** | **122/215 (56.7)** | **254/548 (46.4)** | **0.010** |
| Current smoker, n/N (%) | **51/515 (9.9)** | **44/237 (18.6)** | **0.001** | **22/349 (6.3)** | **73/403 (18.1)** | **<0.001** | **13/215 (6.0)** | **82/537 (15.3)** | **<0.001** |
| Alcohol risk consumption, n/N (%) | 89/458 (19.4) | 30/179 (16.8) | 0.437 | 60/312 (19.2) | 59/325 (18.2) | 0.727 | 42/193 (21.8) | 77/444 (17.3) | 0.189 |
| ADL dependent, n/n (%) | **53/490 (10.8)** | **44/201 (21.9)** | **<0.001** | **25/338 (7.4)** | **72/353 (20.4)** | **<0.001** | **15/202 (7.4)** | **82/489 (16.8)** | **0.002** |
| Myocardial infarction, n/N (%) | **56/520 (10.8)** | **46/247 (18.6)** | **0.003** | **30/354 (8.5)** | **72/413 (17.4)** | **<0.001** | **16/215 (7.4)** | **86/552 (15.6)** | **0.004** |
| Atrial fibrillation, n/N (%) | 57/520 (11.0) | 33/247 (13.4) | 0.335 | **31/354 (8.8)** | **59/413 (14.3)** | **0.019** | 20/215 (9.3) | 70/552 (12.7) | 0.193 |
| Heart failure, n/N (%) | **15/520 (2.9)** | **20/247 (8.1)** | **0.002** | **7/354 (2.0)** | **28/413 (6.8)** | **0.003** | **4/215 (1.9)** | **31/552 (5.6)** | **0.033** |
| Diabetes treatment, n/N (%) | **53/520 (10.2)** | **39/247 (15.8)** | **0.027** | **25/354 (7.1)** | **67/413 (16.2)** | **<0.001** | **13/215 (6.0)** | **79/552 (14.3)** | **0.002** |
| Hypertension treatment, n/N (%) | 190/506 (37.5) | 86/230 (37.4) | 0.967 | **115/345 (33.3)** | **161/391 (41.2)** | **0.029** | **63/212 (29.7)** | **213/524 (40.6)** | **0.006** |
| Stroke, n/N (%) | 48/520 (9.2) | 29/247 (11.7) | 0.281 | **17/354 (4.8)** | **60/413 (14.5)** | **<0.001** | **9/215 (4.2)** | **68/552 (12.3)** | **0.001** |
| Dementia, n/N (%) | **15/519 (2.9)** | **30/239 (12.6)** | **<0.001** | **6/352 (1.7)** | **39/406 (9.6)** | **<0.001** | **2/215 (0.9)** | **43/543 (7.9)** | **0.002** |
| APOE e4, n/N (%) | 141/514 (27.4) | 66/216 (30.6) | 0.393 | 95/353 (26.9) | 112/377 (29.7) | 0.402 | 55/213 (25.8) | 152/517 (29.4) | 0.330 |
| BMI (kg/m^2^), mean ± SD (N) | 26.7± 4.1 (508) | 26.6±4.5 (232) | 0.801 | 26.5± 3.9 (353) | 26.8±4.6 (387) | 0.377 | 26.5±3.7 (213) | 26.6±4.2 (522) | 0.797 |
| SBP (mmHG), mean ± SD (N) | 151±21 (519) | 151±23 (244) | 0.984 | 150±21 (354) | 151±22 (409) | 0.396 | **147±19 (215)** | **152±22 (548)** | **0.005** |
| DBP (mmHG), mean ± SD (N) | 81±10 (519) | 81±11 (244) | 0.322 | 81±10 (354) | 81±11 (409) | 0.708 | 80±9 (215) | 81±11 (547) | 0.221 |
| PEF (% of expected) , mean ± SD (N) | **111±25 (487)** | **103±26 (206)** | **<0.001** | **113±24 (334)** | **104±25 (359)** | **<0.001** | **113±23 (204)** | **106±27 (489)** | **0.001** |
| Gait speed (m/s), mean ± SD (N) | **1.19±0.19 (436)** | **1.13±0.19 (138)** | **0.004** | **1.20±0.18 (302)** | **1.14±0.20 (272)** | **<0.001** | **1.21±0.17 (185)** | **1.16±0.20 (388)** | **0.002** |
| Word fluency, mean ± SD (N) | **21.5±6.5 (508)** | **18.6±7.0 (210)** | **<0.001** | **22.3±6.1 (345)** | **19.1±7.0 (373)** | **<0.001** | **23.1± 6.0 (211)** | **19.8±6.5 (501)** | **<0.001** |
| Free recall, mean ± SD (N) | **7.3±2.0 (508)** | **6.4±2.4 (211)** | **<0.001** | **7.4±1.9 (344)** | **6.7±2.3 (375)** | **<0.001** | **7.6±1.9 (210)** | **6.9±2.1 (502)** | **<0.001** |
| Medications, median [IQR] (N) | **4 [1-6], (510)** | **4 [2-7], (237)** | **<0.001** | **3 [1-5], (349)** | **4 [2-7], (398)** | **<0.001** | **3 [1-5], (213)** | **4 [2-7], (534)** | **<0.001** |
| MADRS, median [IQR] (N) | **4 [2-8], (506)** | **5 [2-11], (205)** | **0.011** | **4 [1-8], (344)** | **5 [2-10], (367)** | **0.002** | **3 [1-7], (210)** | **5 [2-10], (501)** | **0.006** |

P-values are based on logistic regression, bolded numbers are significant at p<0.05; BMI=Body Mass Index, SBP=Systolic Blood Pressure, DBP=Diastolic Blood Pressure, PEF= Peak Expiratory Flow, ADL= Activities of Daily Living, MADRS= Montgomery Åsberg Depression Rating Scale, APOE= Apolipoprotein E
^a^Drop-outs includes participants that have died or emigrated before follow-up, refused participation or contact failure at follow-up, or were excluded due to language difficulties or for technical reasons.
